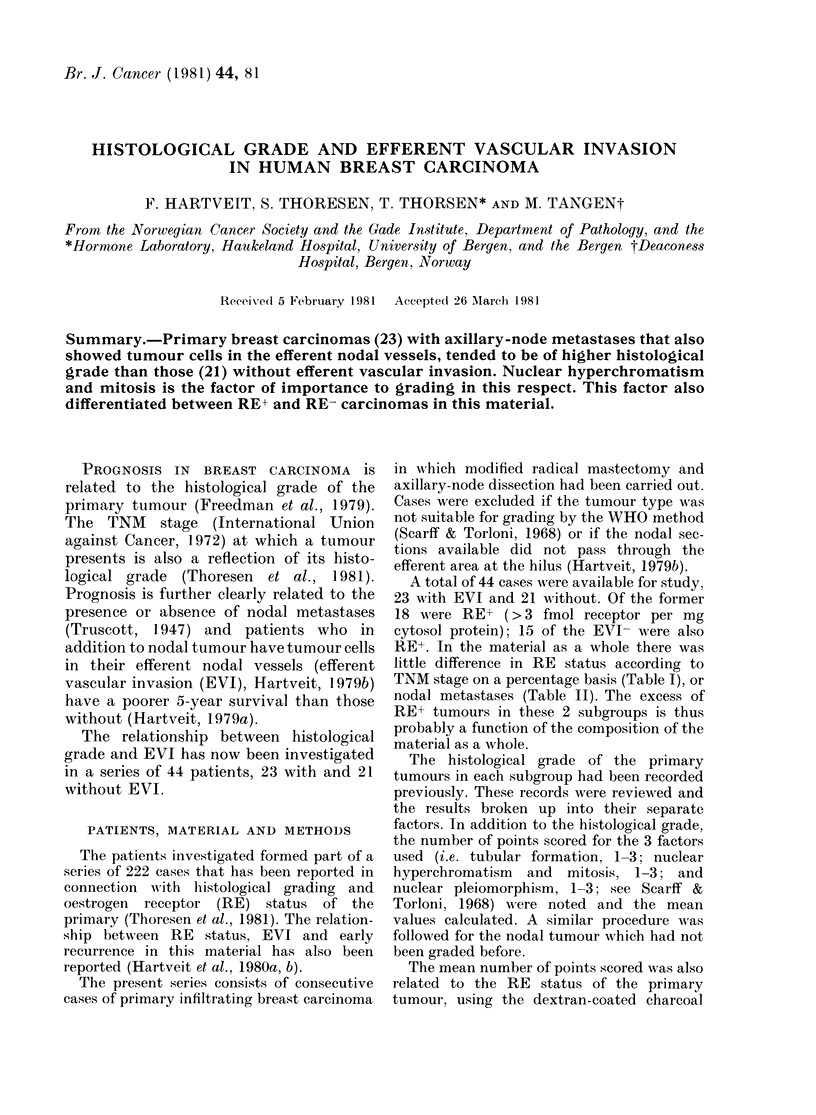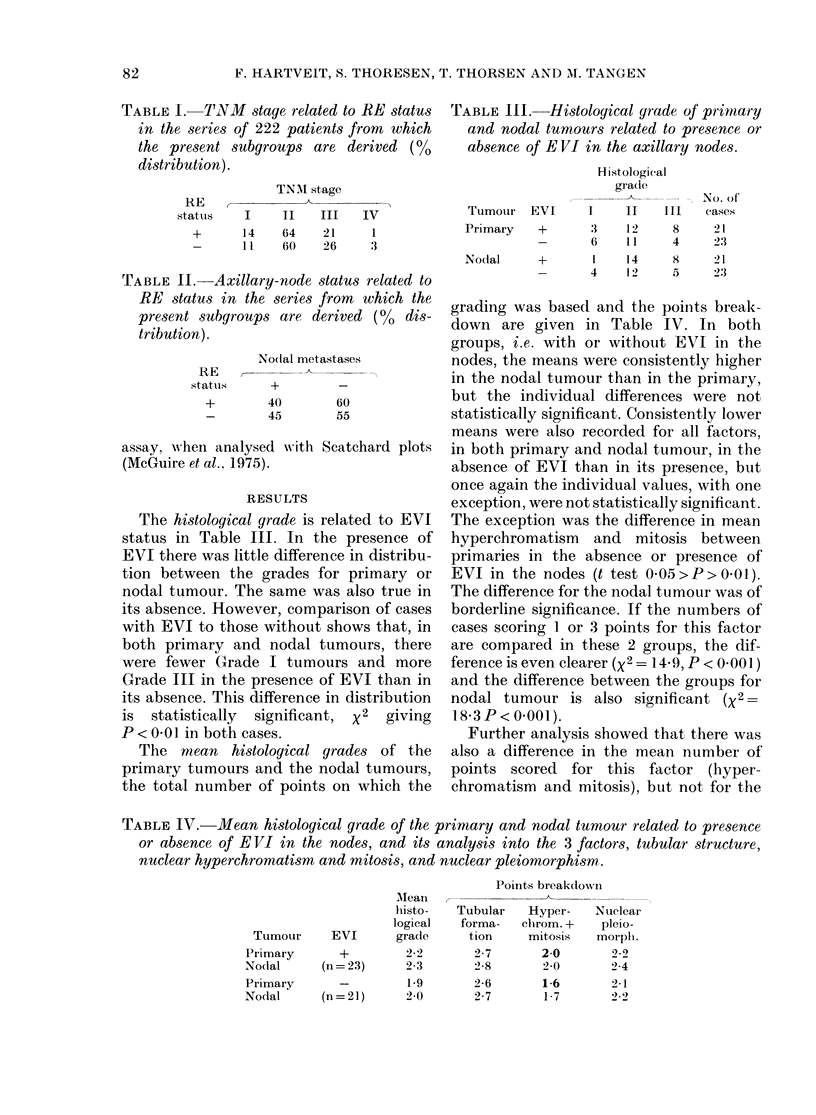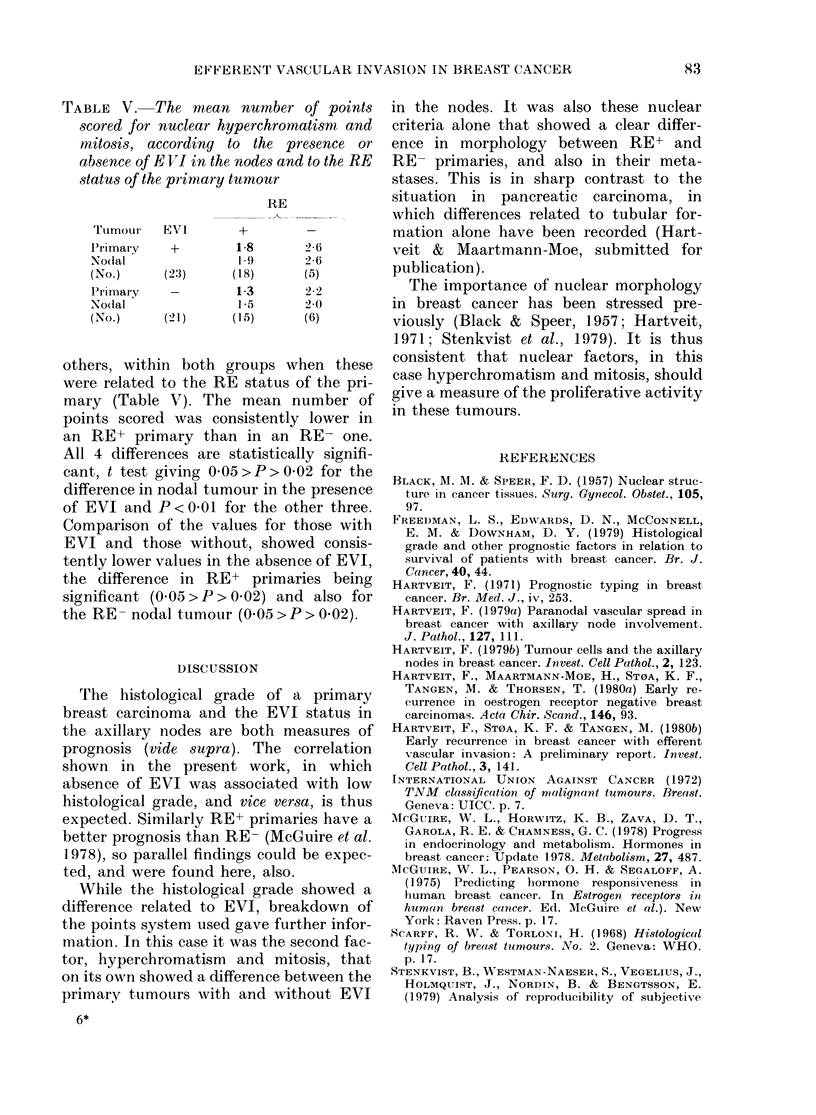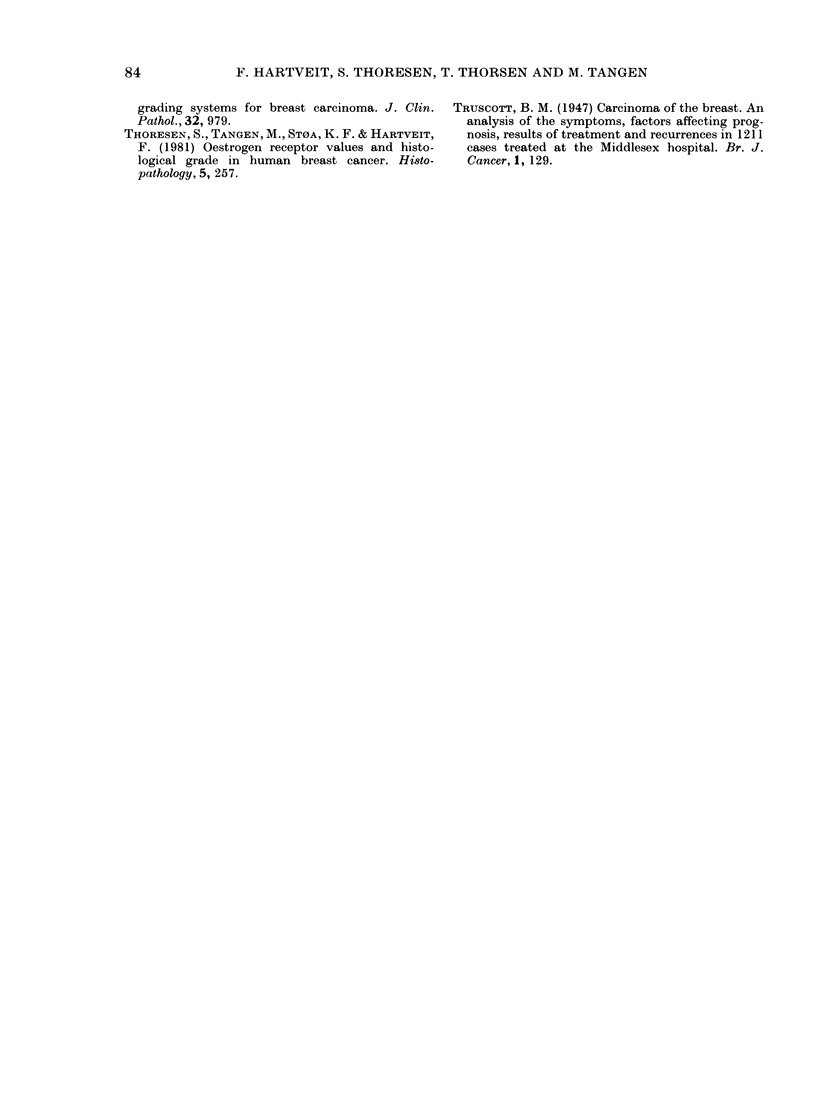# Histological grade and efferent vascular invasion in human breast carcinoma.

**DOI:** 10.1038/bjc.1981.151

**Published:** 1981-07

**Authors:** F. Hartveit, S. Thoresen, T. Thorsen, M. Tangen

## Abstract

Primary breast carcinomas (23) with axillary-node metastases that also showed tumour cells in the efferent nodal vessels, tended to be of higher histological grade than those (21) without efferent vascular invasion. Nuclear hyperchromatism and mitosis is the factor of importance to grading in this respect. This factor also differentiated between RE+ and RE- carcinomas in this material.


*Br. J. Cancer* (1981) **44**, 81

### HISTOLOGICAL GRADE AND EFFERENT VASCULAR INVASION IN HUMAN BREAST CARCINOMA

F. HARTVEIT, S. THORESEN, T. THORSEN* AND M. TANGEN†

*From the Norwegian Cancer Society and the Gade Institute, Department of Pathology, and the *Hormone Laboratory, Haukeland Hospital, University of Bergen, and the Bergen †Deaconess Hospital, Bergen, Norway*

Received 5 February 1981 Accepted 26 March 1981

**Summary.—Primary breast carcinomas (23) with axillary-node metastases that also showed tumour cells in the efferent nodal vessels, tended to be of higher histological grade than those (21) without efferent vascular invasion. Nuclear hyperchromatism and mitosis is the factor of importance to grading in this respect. This factor also differentiated between $RE^+$ and $RE^-$ carcinomas in this material.**

PROGNOSIS IN BREAST CARCINOMA is related to the histological grade of the primary tumour (Freedman *et al.*, 1979). The TNM stage (International Union against Cancer, 1972) at which a tumour presents is also a reflection of its histological grade (Thoresen *et al.*, 1981). Prognosis is further clearly related to the presence or absence of nodal metastases (Truscott, 1947) and patients who in addition to nodal tumour have tumour cells in their efferent nodal vessels (efferent vascular invasion (EVI), Hartveit, 1979*b*) have a poorer 5-year survival than those without (Hartveit, 1979*a*).

The relationship between histological grade and EVI has now been investigated in a series of 44 patients, 23 with and 21 without EVI.

### PATIENTS, MATERIAL AND METHODS

The patients investigated formed part of a series of 222 cases that has been reported in connection with histological grading and oestrogen receptor (RE) status of the primary (Thoresen *et al.*, 1981). The relationship between RE status, EVI and early recurrence in this material has also been reported (Hartveit *et al.*, 1980*a*, *b*).

The present series consists of consecutive cases of primary infiltrating breast carcinoma in which modified radical mastectomy and axillary-node dissection had been carried out. Cases were excluded if the tumour type was not suitable for grading by the WHO method (Scarff & Torloni, 1968) or if the nodal sections available did not pass through the efferent area at the hilus (Hartveit, 1979*b*).

A total of 44 cases were available for study, 23 with EVI and 21 without. Of the former 18 were $RE^+$ ($>3$ fmol receptor per mg cytosol protein); 15 of the $EVI^-$ were also $RE^+$. In the material as a whole there was little difference in RE status according to TNM stage on a percentage basis (Table I), or nodal metastases (Table II). The excess of $RE^+$ tumours in these 2 subgroups is thus probably a function of the composition of the material as a whole.

The histological grade of the primary tumours in each subgroup had been recorded previously. These records were reviewed and the results broken up into their separate factors. In addition to the histological grade, the number of points scored for the 3 factors used (*i.e.* tubular formation, 1–3; nuclear hyperchromatism and mitosis, 1–3; and nuclear pleiomorphism, 1–3; see Scarff & Torloni, 1968) were noted and the mean values calculated. A similar procedure was followed for the nodal tumour which had not been graded before.

The mean number of points scored was also related to the RE status of the primary tumour, using the dextran-coated charcoal

TABLE I.—*TNM stage related to RE status in the series of 222 patients from which the present subgroups are derived (% distribution).*

| RE status | TNM stage I | II | III | IV |
|---|---|---|---|---|
| + | 14 | 64 | 21 | 1 |
| – | 11 | 60 | 26 | 3 |

TABLE II.—*Axillary-node status related to RE status in the series from which the present subgroups are derived (% distribution).*

| RE status | Nodal metastases + | – |
|---|---|---|
| + | 40 | 60 |
| – | 45 | 55 |

assay, when analysed with Scatchard plots (McGuire *et al.*, 1975).

### RESULTS

The *histological grade* is related to EVI status in Table III. In the presence of EVI there was little difference in distribution between the grades for primary or nodal tumour. The same was also true in its absence. However, comparison of cases with EVI to those without shows that, in both primary and nodal tumours, there were fewer Grade I tumours and more Grade III in the presence of EVI than in its absence. This difference in distribution is statistically significant, $\chi^2$ giving $P < 0{\cdot}01$ in both cases.

The *mean histological grades* of the primary tumours and the nodal tumours, the total number of points on which the grading was based and the points breakdown are given in Table IV. In both groups, *i.e.* with or without EVI in the nodes, the means were consistently higher in the nodal tumour than in the primary, but the individual differences were not statistically significant. Consistently lower means were also recorded for all factors, in both primary and nodal tumour, in the absence of EVI than in its presence, but once again the individual values, with one exception, were not statistically significant. The exception was the difference in mean hyperchromatism and mitosis between primaries in the absence or presence of EVI in the nodes ($t$ test $0{\cdot}05 > P > 0{\cdot}01$). The difference for the nodal tumour was of borderline significance. If the numbers of cases scoring 1 or 3 points for this factor are compared in these 2 groups, the difference is even clearer ($\chi^2 = 14{\cdot}9, P < 0{\cdot}001$) and the difference between the groups for nodal tumour is also significant ($\chi^2 = 18{\cdot}3\, P < 0{\cdot}001$).

TABLE III.—*Histological grade of primary and nodal tumours related to presence or absence of EVI in the axillary nodes.*

| Tumour | EVI | Histological grade I | II | III | No. of cases |
|---|---|---|---|---|---|
| Primary | + | 3 | 12 | 8 | 21 |
| | – | 6 | 11 | 4 | 23 |
| Nodal | + | 1 | 14 | 8 | 21 |
| | – | 4 | 12 | 5 | 23 |

Further analysis showed that there was also a difference in the mean number of points scored for this factor (hyperchromatism and mitosis), but not for the

TABLE IV.—*Mean histological grade of the primary and nodal tumour related to presence or absence of EVI in the nodes, and its analysis into the 3 factors, tubular structure, nuclear hyperchromatism and mitosis, and nuclear pleiomorphism.*

| Tumour | EVI | Mean histological grade | Points breakdown: Tubular formation | Hyperchrom. + mitosis | Nuclear pleiomorph. |
|---|---|---|---|---|---|
| Primary | + | 2·2 | 2·7 | **2·0** | 2·2 |
| Nodal | (n = 23) | 2·3 | 2·8 | 2·0 | 2·4 |
| Primary | – | 1·9 | 2·6 | **1·6** | 2·1 |
| Nodal | (n = 21) | 2·0 | 2·7 | 1·7 | 2·2 |

TABLE V.—*The mean number of points scored for nuclear hyperchromatism and mitosis, according to the presence or absence of EVI in the nodes and to the RE status of the primary tumour*

| | | RE | |
|---|---|---|---|
| Tumour | EVI | + | − |
| Primary | + | **1·8** | 2·6 |
| Nodal | | 1·9 | 2·6 |
| (No.) | (23) | (18) | (5) |
| Primary | − | **1·3** | 2·2 |
| Nodal | | 1·5 | 2·0 |
| (No.) | (21) | (15) | (6) |

others, within both groups when these were related to the RE status of the primary (Table V). The mean number of points scored was consistently lower in an $RE^+$ primary than in an $RE^-$ one. All 4 differences are statistically significant, *t* test giving $0{\cdot}05 > P > 0{\cdot}02$ for the difference in nodal tumour in the presence of EVI and $P < 0{\cdot}01$ for the other three. Comparison of the values for those with EVI and those without, showed consistently lower values in the absence of EVI, the difference in $RE^+$ primaries being significant ($0{\cdot}05 > P > 0{\cdot}02$) and also for the $RE^-$ nodal tumour ($0{\cdot}05 > P > 0{\cdot}02$).

### DISCUSSION

The histological grade of a primary breast carcinoma and the EVI status in the axillary nodes are both measures of prognosis (*vide supra*). The correlation shown in the present work, in which absence of EVI was associated with low histological grade, and *vice versa*, is thus expected. Similarly $RE^+$ primaries have a better prognosis than $RE^-$ (McGuire *et al.* 1978), so parallel findings could be expected, and were found here, also.

While the histological grade showed a difference related to EVI, breakdown of the points system used gave further information. In this case it was the second factor, hyperchromatism and mitosis, that on its own showed a difference between the primary tumours with and without EVI in the nodes. It was also these nuclear criteria alone that showed a clear difference in morphology between $RE^+$ and $RE^-$ primaries, and also in their metastases. This is in sharp contrast to the situation in pancreatic carcinoma, in which differences related to tubular formation alone have been recorded (Hartveit & Maartmann-Moe, submitted for publication).

The importance of nuclear morphology in breast cancer has been stressed previously (Black & Speer, 1957; Hartveit, 1971; Stenkvist *et al.*, 1979). It is thus consistent that nuclear factors, in this case hyperchromatism and mitosis, should give a measure of the proliferative activity in these tumours.

### REFERENCES

BLACK, M. M. & SPEER, F. D. (1957) Nuclear structure in cancer tissues. *Surg. Gynecol. Obstet.*, **105**, 97.

FREEDMAN, L. S., EDWARDS, D. N., MCCONNELL, E. M. & DOWNHAM, D. Y. (1979) Histological grade and other prognostic factors in relation to survival of patients with breast cancer. *Br. J. Cancer*, **40**, 44.

HARTVEIT, F. (1971) Prognostic typing in breast cancer. *Br. Med. J.*, iv, 253.

HARTVEIT, F. (1979*a*) Paranodal vascular spread in breast cancer with axillary node involvement. *J. Pathol.*, **127**, 111.

HARTVEIT, F. (1979*b*) Tumour cells and the axillary nodes in breast cancer. *Invest. Cell Pathol.*, **2**, 123.

HARTVEIT, F., MAARTMANN-MOE, H., STØA, K. F., TANGEN, M. & THORSEN, T. (1980*a*) Early recurrence in oestrogen receptor negative breast carcinomas. *Acta Chir. Scand.*, **146**, 93.

HARTVEIT, F., STØA, K. F. & TANGEN, M. (1980*b*) Early recurrence in breast cancer with efferent vascular invasion: A preliminary report. *Invest. Cell Pathol.*, **3**, 141.

INTERNATIONAL UNION AGAINST CANCER (1972) *TNM classification of malignant tumours. Breast.* Geneva: UICC. p. 7.

MCGUIRE, W. L., HORWITZ, K. B., ZAVA, D. T., GAROLA, R. E. & CHAMNESS, G. C. (1978) Progress in endocrinology and metabolism. Hormones in breast cancer: Update 1978. *Metabolism*, **27**, 487.

MCGUIRE, W. L., PEARSON, O. H. & SEGALOFF, A. (1975) Predicting hormone responsiveness in human breast cancer. In *Estrogen receptors in human breast cancer*. Ed. McGuire *et al.*). New York: Raven Press. p. 17.

SCARFF, R. W. & TORLONI, H. (1968) *Histological typing of breast tumours. No.* 2. Geneva: WHO. p. 17.

STENKVIST, B., WESTMAN-NAESER, S., VEGELIUS, J., HOLMQUIST, J., NORDIN, B. & BENGTSSON, E. (1979) Analysis of reproducibility of subjective grading systems for breast carcinoma. *J. Clin. Pathol.*, **32**, 979.

THORESEN, S., TANGEN, M., STØA, K. F. & HARTVEIT, F. (1981) Oestrogen receptor values and histological grade in human breast cancer. *Histopathology*, **5**, 257.

TRUSCOTT, B. M. (1947) Carcinoma of the breast. An analysis of the symptoms, factors affecting prognosis, results of treatment and recurrences in 1211 cases treated at the Middlesex hospital. *Br. J. Cancer*, **1**, 129.